# Microbiota promotes recruitment and pro-inflammatory response of caecal macrophages during *E. tenella* infection

**DOI:** 10.1186/s13099-023-00591-8

**Published:** 2023-12-14

**Authors:** F. Tomal, A. Sausset, Y. Le Vern, L. Sedano, C. Techer, S. Lacroix-Lamandé, F. Laurent, A. Silvestre, F. I. Bussière

**Affiliations:** 1 INRAE, Université de Tours, UMR ISP, 37380 Nouzilly, France; 2 MixScience, 35170 Bruz, France

**Keywords:** *Eimeria tenella*, Macrophages, Microbiota, Inflammation, Immune response, Germ-free, Chicken

## Abstract

**Background:**

*Eimeria genus* belongs to the apicomplexan parasite phylum and is responsible for coccidiosis, an intestinal disease with a major economic impact on poultry production. *Eimeria tenella* is one of the most virulent species in chickens. In a previous study, we showed a negative impact of caecal microbiota on the physiopathology of this infection. However, the mechanism by which microbiota leads to the physiopathology remained undetermined. Macrophages play a key role in inflammatory processes and their interaction with the microbiota during *E. tenella* infection have never been investigated. We therefore examined the impact of microbiota on macrophages during *E. tenella* infection. Macrophages were monitored in caecal tissues by immunofluorescence staining with KUL01 antibody in non-infected and infected germ-free and conventional chickens. Caecal cells were isolated, stained, analyzed and sorted to examine their gene expression using high-throughput qPCR.

**Results:**

We demonstrated that microbiota was essential for caecal macrophage recruitment in *E. tenella* infection. Furthermore, microbiota promoted a pro-inflammatory transcriptomic profile of macrophages characterized by increased gene expression of *NOS2, ACOD1, PTGS2, TNFα, IL1β, IL6, IL8L1, IL8L2* and *CCL20* in infected chickens. Administration of caecal microbiota from conventional chickens to germ-free infected chickens partially restored macrophage recruitment and response.

**Conclusions:**

Taken together, these results suggest that the microbiota enhances the physiopathology of this infection through macrophage recruitment and activation. Consequently, strategies involving modulation of the gut microbiota may lead to attenuation of the macrophage-mediated inflammatory response, thereby limiting the negative clinical outcome of the disease.

**Supplementary Information:**

The online version contains supplementary material available at 10.1186/s13099-023-00591-8.

## Introduction

Coccidiosis is caused by an apicomplexan protozoan parasite of the *genus Eimeria*. This parasite colonizes the intestines of various animal species and is host-specific. Seven *Eimeria* species and three cryptic species have been described in chickens, each colonizing a preferential region of the intestine. The overall cost of prophylaxis and losses due to *Eimeria* infection have significant economic impact, estimated at around $13 billion (USD) per year in the poultry industry [1]. Moreover, modern poultry farming favors infections as birds are reared in very large numbers in confined areas, with high density of individuals [2].

*Eimeria tenella* is one of the most pathogenic species that can cause hemorrhagic diarrhoea, and death in the most severe cases. This parasite replicates in the epithelial cells of the caeca, leading to acute inflammation with an increase in the number of several types of immune cells in the caecal tissue [3]. Parasite replication and the acute inflammatory response associated with infection lead to the genesis of caecal lesions. This excessive inflammatory response is linked to the infiltration of inflammatory cells and the subsequent release of pro-inflammatory mediators such as *nitric oxide* (*NO), prostaglandins, interferon (IFN)γ, interleukin (IL)1β, IL6* and *IL17* [4, 5]. Among the inflammatory cells at the site of infection, macrophages were found in large numbers after primary infection with *E. tenella* [6] and also during infection with other species such as *E. bovis* [7]. Macrophages are innate immune cells that play a key role in the first line of defense against pathogens, regulating inflammation. In vitro, exposure of the HTC avian macrophage cell line to *E. tenella* sporozoites induces expression of various cytokines, *IL1β*, *IL6* and *IL17* and chemokines, *macrophage inflammatory protein-1* (*MIP1β)*, *K203 (CCL4), ah221 (Chemokine (C–C motif) ligand 17 (CCL17)* and *K60 (IL8L1)* [8]. More recently, in the avian macrophage cell line, HD11 cells, infected with *E. tenella* sporozoites, increased gene expression of pathogen recognition receptors (PRR), metalloproteinases, chemokines and genes related to interferon stimulation was observed [9]. All these studies were carried out in vitro and, although they provide valuable information on the macrophage response to *E. tenella* infection, their response at the site of infection remains to be studied.

*E. tenella* replicates in the caeca, the richest and most diverse bacterial segment of the intestinal tract [10, 11]. Infection with *E. tenella* leads to changes in the diversity and composition of the microbiota associated with an increase in *Enterobacteriaceae* while non-pathogenic bacteria such as *Lactobacillus* and *Faecalibacterium* decrease [12–15]. In addition, the microbiota is essential for the development and maturation of the immune system [16]. In previous studies, we reported that in *E. tenella* infection, the caecal microbiota plays a role in parasite development [17], lesion formation, inflammatory response and caecal barrier integrity [18] but the mechanism leading to the pathology remains to be investigated.

In the present study, we sought to understand the impact of microbiota on macrophage recruitment and response during *E. tenella* infection using conventional and germ-free (GF) chickens. These results suggest a crucial role for the microbiota in macrophage recruitment and pro-inflammatory response. Consequently, macrophages are suspected of playing a major role in the physiopathology of this infection. In the future, our work opens up a new avenue of research aimed at optimizing the composition of the microbiota to limit macrophage-mediated inflammatory response and consequently, clinical outcomes.

## Materials and methods

### Ethical statement

Animal experiments were carried out in accordance with the French legislation (Décret: 2001‐464 29/05/01) and the EEC regulation (86/609/CEE) and approved by the Centre Val de Loire ethics committee (CEEA VdL n°19): 2018‐04‐26 (APAFIS N°13904).

### Animals and infection

Conventional and GF Ross PM3 broilers were hatched in the Infectiology of Farm, Model and Wildlife Animals Facility (PFIE, Centre INRAE Val De Loire; https://doi.org/10.15454/1.5572352821559333E12; member of the National Infrastructure EMERG’IN) as described in [19]. Briefly, Ross PM3 eggs from two French farms were collected, decontaminated with a 1.5% peracetic acid solution (1.5% Divosan Plus VT53, Johnson Diversey, France). Eggs were then incubated, decontaminated a second time and placed in a hatching incubator for conventional chicks or an isolator for GF animals. All animals received no vaccination and were fed irradiated feed (SAFE^®^) without any additives. Two experimental replicates were performed using 5 groups of chickens (non-infected: conventional or GF and, infected: conventional, GF or GF + microbiota; Table 1) and data were combined together. Two-week-old chickens were orally infected with 10,000 sporulated oocysts of the *Eimeria tenella* INRAE PAPt36 strain (*Et*-INRAE) strain (n = 31 for conventional and n = 21 for GF chickens). A group of GF-infected chickens received a microbiota from healthy conventional animals at 4 days by oral gavage of caecal contents of 4-week-old chickens from our animal facility (GF + microbiota group; *n* = 24). Thirty-six conventional and 22 GF non-infected chickens were used as control respectively for flow cytometry analysis. Among these animals, KUL01^+^ cells were sorted to obtain samples from 8 conventional and GF non-infected, 11 conventional and GF and 9 GF + microbiota chickens for transcriptomic analysis. Bacteriological controls were performed as described in [19]. Animals were confirmed to be bacteria-free while conventional chickens developed a microbiota. At 7 days pi, chickens were euthanized by electronarcosis and caeca were collected.

**Table 1:** Experimental groups of chickens

Groups	Microbiota	Infection
Conventional non-infected	+	−
Germ-Free non-infected	−	−
Conventional infected	+	+
Germ-Free infected	−	+
Germ-Free infected + Microbiota	Added at 4 dpi	+

In these experiments, conventional non-infected, GF non-infected, conventional infected, GF-infected, GF-infected receiving a microbiota from conventional chickens at 4 days post-infection were studied for macrophage recruitment and response

### Isolation of caecal cells

Caeca were harvested, opened longitudinally and washed three times with HBSS. The mucosa was scraped with a scalpel and placed in 25 mL in Dulbecco’s Modified Eagle Medium: Nutrient Mixture F-12 with 15 mM HEPES, L-Glutamine (DMEM-F12, Gibco, Life Technologies Limited, Paisley, UK), 10% of fetal bovine serum (FBS, Dutscher, Bernolsheim, France) and 1% of penicillin–streptomycin (P/S, Cytiva, Hyclone, Laboratories, South Logan, UT, USA) with 0.5 mg/mL collagenase H (1 mg/mL, Roche, Basel, Switzerland) at 37 °C for 30 min under continuous agitation. The cell suspension was filtered through a 50 µm filter. After centrifugation 900 g for 10 min, the cells were resuspended and added to 5 mL of lymphocyte separation medium (density 1.077) (Eurobio Scientific, Les Ulis, France). After centrifugation for 30 min at 700 g, the leukocyte ring was collected and washed twice in 15 mL of DMEM-F12 with 10% FBS and 1% P/S. Centrifugation was performed for 10 min at 900 g. The cell pellet was resuspended in a minimum volume and cell viability was assessed by Trypan blue. Only viable cells were counted. Cells (2 × 10^6^) were placed in a 96-well plate, centrifuged at 900 g for 5 min at 4 °C.

### Flow cytometry analysis and cell sorting

For flow cytometry and cell sorting, cells were stained with Zombie Aqua™ (BioLegend, San Diego, CA, USA), mouse anti-chicken MHCII-Alexa fluor 488, mouse anti-chicken KUL01-PE and mouse anti-chicken CD45-APC (SouthernBiotech, Birmingham, AL, USA) in PBS with 2% FBS and 2 mM EDTA and incubated in the dark at 4°C for 30 min. Cells were washed twice. Cell suspensions were filtered through a 60 µm filter, analyzed on LSR Fortessa X-20 (BD Biosciences, Franklin Lakes, NJ, USA) and sorted using MoFlo Astrios EQ, Summit software 6.3 (Beckman Coulter, Brea, CA, USA). Flow cytometry data were analyzed using Kaluza Analysis software 2.1 (Beckman Coulter). Debris were excluded from the analysis.

### Immunofluorescence staining

Caecal tissue samples were snap flash-frozen in optimal cutting temperature compound (OCT). Embedded frozen tissues were sliced with Leica CM3050 S Cryostat (Leica, Wetzlar, Germany) and fixed in ice cold 50% ethanol-50% acetone. Samples were permeabilized and Fc receptors were blocked using a solution of PBS, 0.01% Triton X-100, 0.5% BSA, 5% heat inactivated horse and goat sera for 30 min at room temperature. After washing, slides were stained for 2 h at room temperature, using a primary mouse anti-chicken-KUL01 antibody (Biorad, Hercules, CA, USA). After washing, Alexa 594 goat anti-mouse secondary antibody (Invitrogen, Waltham, MA, USA) was added. Cell nuclei were counterstained with 1 µg/mL DAPI (ThermoFisher Scientific) for 10 min at room temperature and mounted using Permafluor™ (Epredia, Kalamazoo, MI, USA). Slides were observed under a Zeiss Axiovert 200M microscope (Zeiss, Jena, Germany) at 5 × magnificence and images were acquired with Zen imaging software (Zeiss, Jena, Germany).

### Reverse transcription and preamplification

Reverse transcription was performed on 3000 sorted CD45^+^ KUL01^+^ cells as previously described [20]. One µL of 100 µM forward and reverse primers (Additional file 4: Table S1) were pooled into a unique tube with Tris EDTA (TE) buffer (Invitrogen, Waltham, MA, USA) to a final volume of 200 µL. A preamplication mix was performed using the previous primer mix and Fluidigm PreAmp Mastermix (Standard BioTools, San Francisco, CA, USA). In a 96-well PCR plate, 3.75 µL of preamplication mix was added to 1.25 µL of cDNA samples. Preamplication was carried out under the following conditions: 2 min at 95 °C, 14 cycles at 95 °C for 15s and 60 °C for 4 min. A clean-up step using Exonuclease I (New England Biolabs, Ipswich, MA, USA) was used to remove unincorporated primers as follows: 2 µL of Exonuclease I was added to each sample and incubated at 37 °C for 30 min and 80 °C for 15 min. Samples were diluted using TE buffer (1:5) and stored at – 20 °C.

### High-throughput qPCR

Quantitative PCR was performed in the BioMark™ HD instrument with a 96.96 Dynamic Array™ IFC for Gene Expression (Standard BioTools, San Francisco, CA, USA). The sample mix was prepared by mixing 2.5 µL 2X SSoFast™ EvaGreen^®^ Supermix with low ROX (Biorad, Hercules, CA, USA) and 0.25 µL 20X DNA Binding Dye (Standard BioTools, San Francisco, CA, USA). Assay mix was prepared by mixing 2.5 µL 2X Assay Loading Reagent (Standard BioTools, San Francisco, CA, USA), 2.25 µL of 1X DNA Suspension Buffer (TEKnova, Hollister, CA, USA). The plate was run through the BioMark™ HD according to the manufacturer’s instructions. Real-Time PCR Analysis software (Standard BioTools, San Francisco, CA, USA) was used to visualize results and extract data.

### Heatmap and differential expression

2^−Delta Ct^ were calculated for each gene using the mean of three *Gallus gallus* housekeeping genes: *g10, gapdh* and *β-2-microglobuline*. A heatmap analysis was performed using the R package ComplexHeatmap [21] to obtain a global view of gene expressions. Gene expression values were normalized with a Z-score approach and scaled by genes. A PAM (Partitioning Around Medoids) was performed using hierarchical clustering to identify clusters. Differential expression (DE) was performed with EnhancedVolcano R package [22]. The thresholds used for DE was fold change > 2 or < − 2 and *p value* adjusted for multiple hypothesis testing was determined using the Benjamini–Hochberg method, with a threshold < 0.05. Comparisons were established as a ratio. Infected conventional and GF chickens were compared to their respective non-infected group. At 7 days pi, conventional were compared to GF and GF + microbiota. A comparison between GF-infected + microbiota and GF-infected chickens was also performed.

### Multivariate analysis

To investigate the influence of microbiota on the transcriptomic profile of macrophages from non-infected and infected chickens, PCA (Principal Component Analysis) were performed. Values were transformed into log10 and PCA was generated using FactoMineR [23] and ggpubr R packages [24].

### Statistical analysis

For flow cytometry data, statistical analysis was performed using a Kruskall-Wallis combined with a Dunn’s multiple comparisons post-test (GraphPad Prism^®^ 6; GraphPad Software Inc., La Jolla, CA, USA). For multivariate analysis, statistical differences between groups were assessed using a pairwise PERMANOVA (Permutational multivariate analysis of variance) test with 999 permutations, Vegan [25] and pairwiseAdonis [26] R packages. All R packages were run from RStudio software, R version 4.1.0.

## Results

### Microbiota promotes macrophage infiltration in *E. tenella* infection

**Fig. 1:**
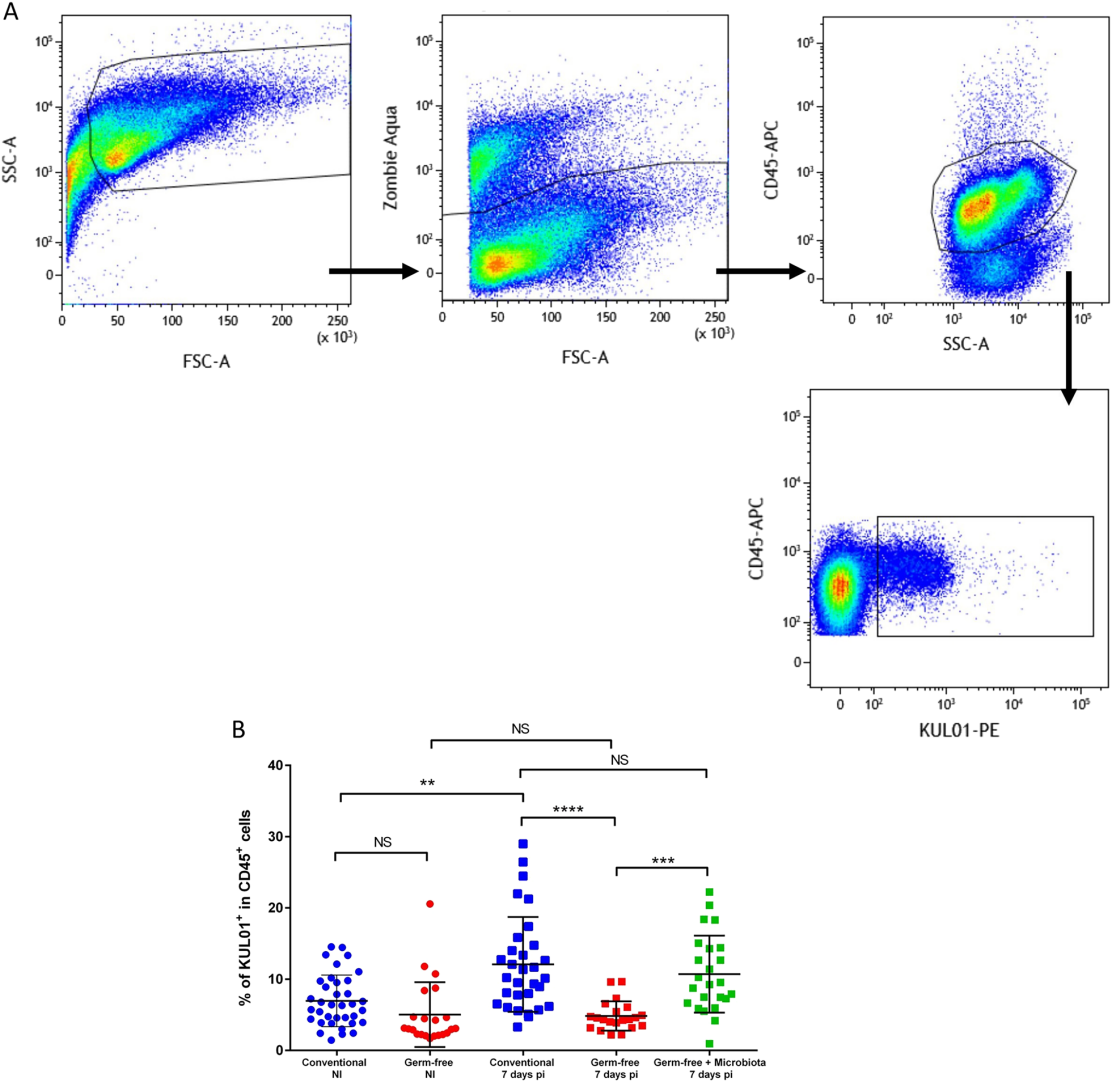
Microbiota promotes macrophage recruitment during *E. tenella* infection. Caecal cells were isolated from 3 weeks old Ross PM3 chickens and stained with Zombie Aqua™, CD45-APC and KUL01-PE. A gate based in FSH-A and SSC-A was placed to remove debris. Live cells were selected using a live/dead marker, Zombie Aqua™. Leukocytes were stained with CD45 antibody and macrophage cells were identified using the KUL01 (MRC1L-B) marker (**A**). Percentage of KUL01^+^ cells within the leukocyte population was determined in conventional (blue), GF chickens (red) in non-infected (NI) and at 7 days pi. In green, GF-infected chickens received a microbiota from conventional healthy chickens at 4 days pi (**B**). Kruskal Wallis test followed by Dunn test. (**P ≤ 0.01; ***P ≤ 0.001; ****P ≤ 0.0001; NS: non-significant)

**Fig. 2:**
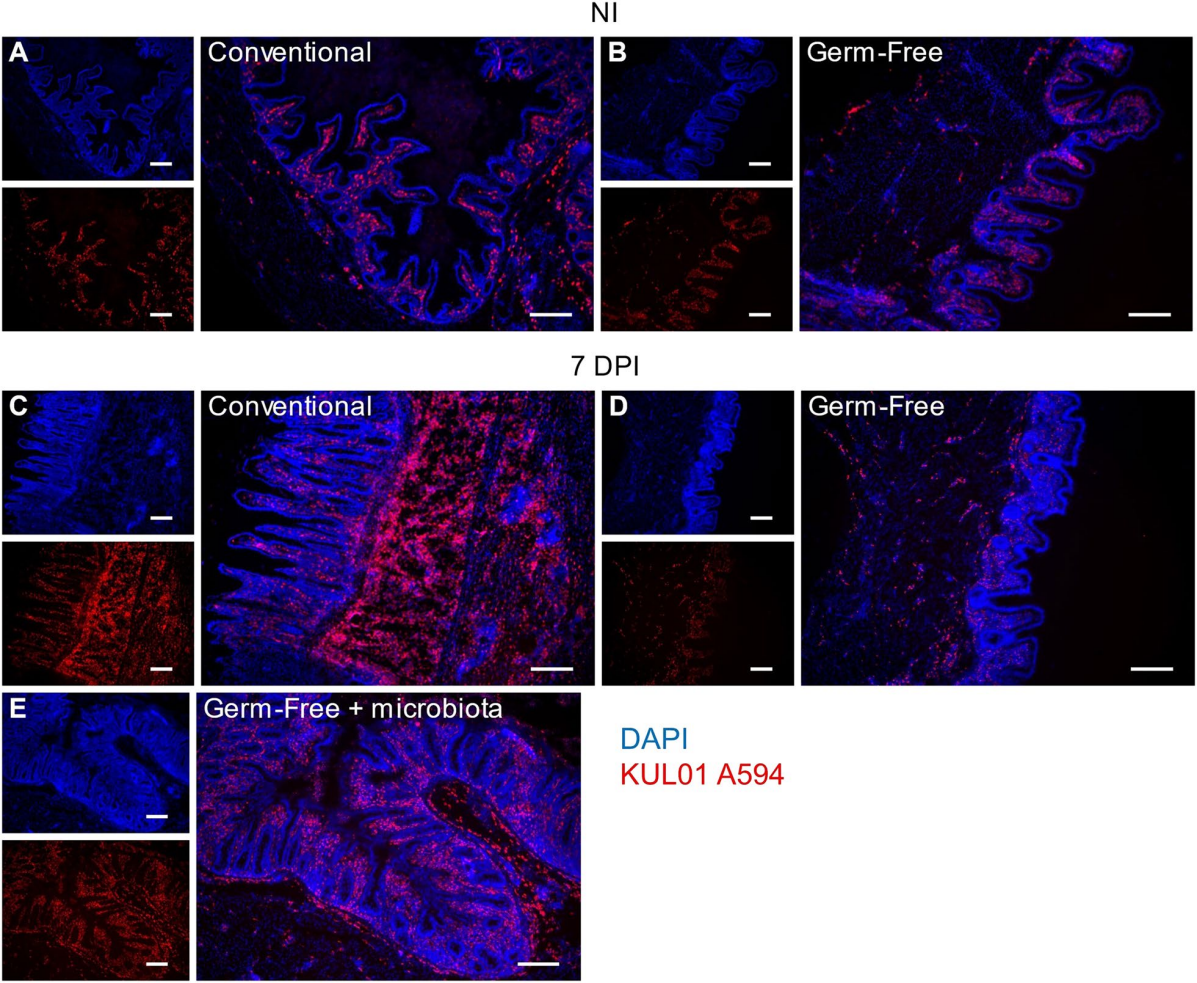
Localization of KUL01^+^ cells during *E. tenella* infection in caeca from conventional and GF chickens. Imbedded frozen OCT caecal sections were stained with DAPI (Blue) and KUL01-Alexa 594 (Red). Non-infected and 7 days pi conventional (**A** and **C**), 7 days pi GF (**B** and **D**), 7 days pi GF chickens receiving a microbiota from healthy conventional chickens at 4 days pi (**E**). Magnification 5X and scale bar indicated 100µm

Chickens were infected with 10 000 *E. tenella* oocysts to obtain the same parasite load in conventional and GF chickens at 7 days pi as described previously [17]. We have previously shown that microbiota promotes immune cell recruitment and excessive inflammation during *E. tenella* infection [18]. We therefore investigated the recruitment of macrophages defined as KUL01^+^ (MRC1L-B) cells to the site of infection in the presence and absence of microbiota. First, we identified macrophages by flow cytometry. After gating live leukocytes (CD45^+^; Zombie Aqua™-negative), a single population of KUL01^+^ within leukocytes was observed (Fig. 1A). Subsets of chicken splenic macrophages have been described using MHCII and KUL01 antibodies [27]. Here, we confirmed the identification of these subsets in the spleen but not in the caeca, in which a single population of MHCII^+^ KUL01^+^ macrophages was observed (Additional file 1: Figure S1). We then determined the percentage of KUL01^+^ cells within leukocytes in non-infected and in infected conventional and GF chickens (Fig. 1B). In contrast to mammals [28–30], at homeostasis we observed no significant difference by flow cytometry and by visualization of macrophages between conventional and GF non-infected chickens (median 6.4% in conventional *versus* 3.1% in GF chickens; Figs. 1B, 2A and B). At 7 days pi, an increase in the percentage of macrophages was observed only in conventional chickens (median 10.2% in conventional *versus* 4.4% in GF-infected chickens) indicating that, surprisingly, the presence of microbiota was essential for the recruitment of macrophages (Figs. 1B, 2C and D). When GF-infected chickens received a microbiota from healthy conventional chickens at 4 days pi, a significant increase in the percentage of macrophages was observed at 7 days pi to a level similar to that observed in conventional infected chickens (median 10.2% in infected conventional chickens *versus* 9.4% in infected GF chickens receiving a microbiota). Immunostaining of caecal tissues with the KUL01 antibody confirmed these observations (Fig. 2). The increase in macrophage recruitment coincides with an alteration in barrier integrity. Indeed, at 3.5 days pi, no increase in macrophages was observed and intestinal barrier integrity has been shown to be maintained [31]. Conversely, at day 5.5 pi, when the intestinal epithelial barrier is impaired, macrophages were found to be more numerous in the caecal tissue (data not shown).

### Microbiota-independent macrophage response to *E. tenella* infection

**Fig. 3:**
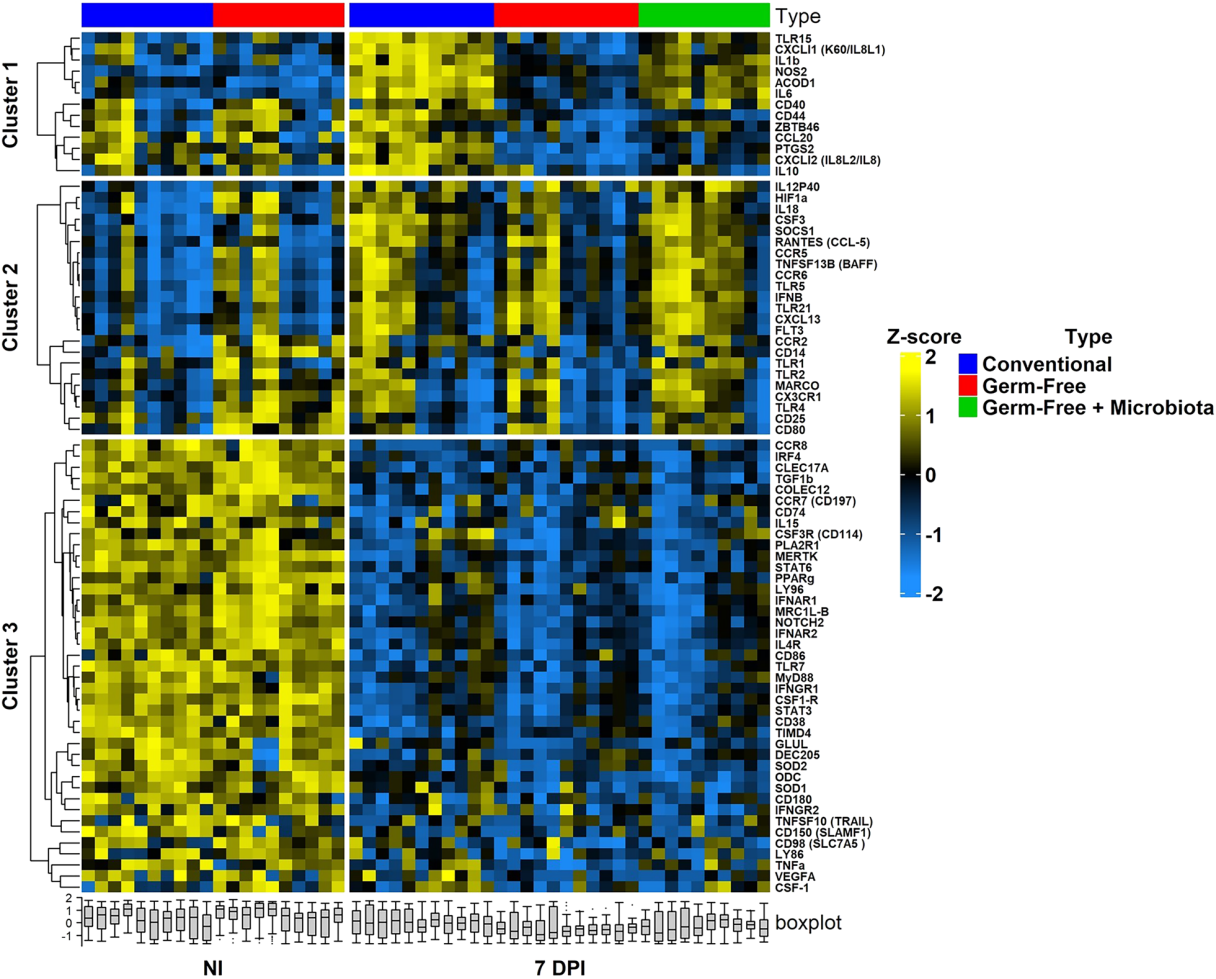
Heatmap of transcriptomic profiles of sorted KUL01^+^ caecal cells from non-infected and 7 days infected conventional and GF chickens. NI and 7 DPI represent non-infected and 7 days pi chickens, respectively. Conventional chickens are indicated in blue, GF chickens in red and GF chickens that received a conventional microbiota in green. Delta Ct gene expression was normalized using a Z-score for each gene. Gradient color from blue to yellow indicated low level and high-level expression respectively. A hierarchical clustering was performed following Z-score for each gene and clusters are indicated in the left of the heatmap (n ≥ 8 chickens / group)

**Fig. 4:**
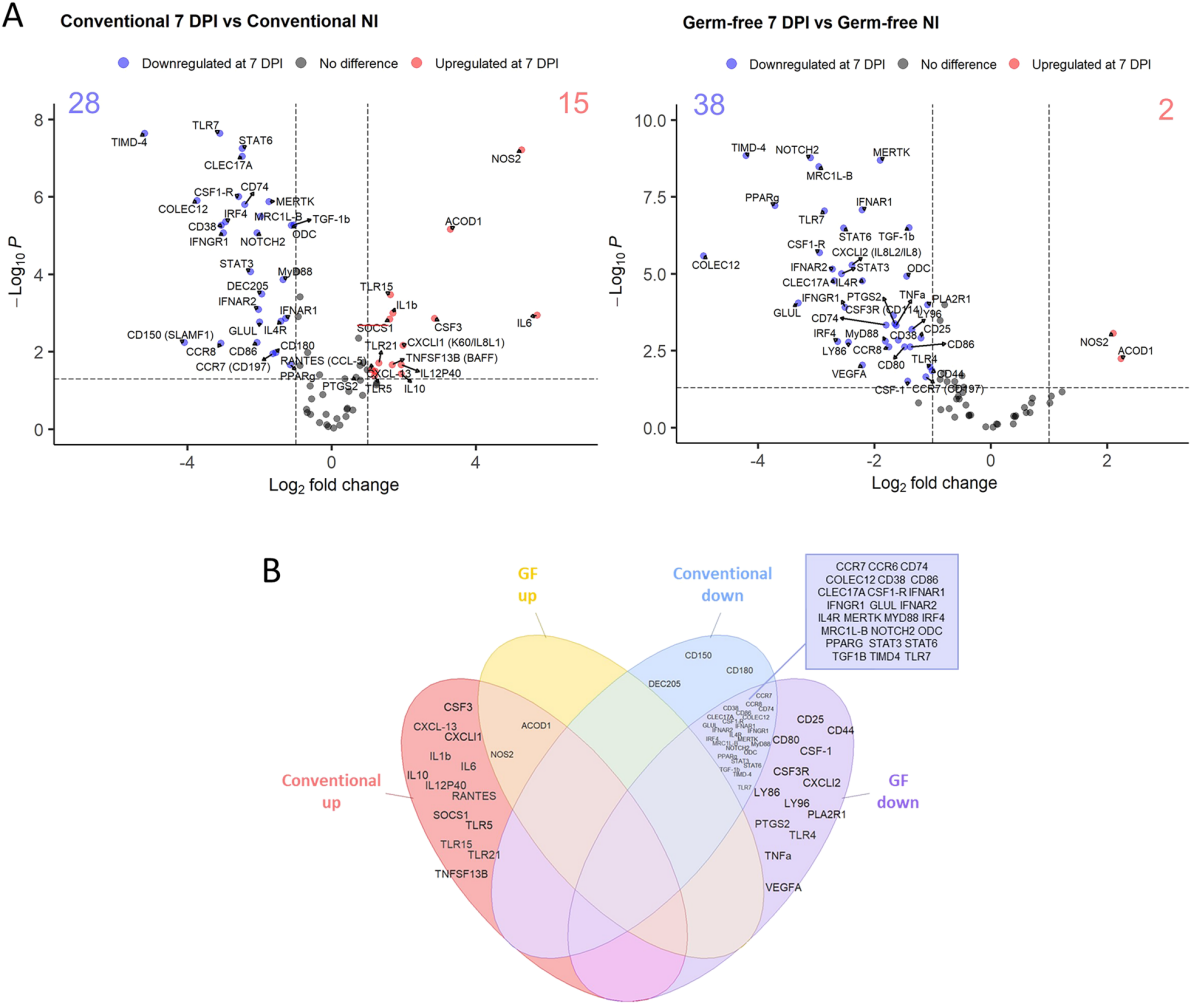
*E. tenella* infection leads to a different macrophage response between conventional and GF chickens. **A** Differential gene expression was determined for 7 days post infected conventional *versus* (*vs*) non-infected conventional (left) and 7 days post infected GF *vs* non-infected GF (right) on volcano plots. Scattered dots represent each gene. Only significantly different gene expression (P < 0.05) and fold change > 2 or < − 2 are indicated. These thresholds are indicated with dashed lines on the plots. PTGS2 is underlined in red indicating that close significant up regulation. (**B**) Venn diagram showing differences and similarities in upregulated and downregulated genes in conventional and GF-infected chickens

To determine the macrophage response to infection, macrophages (CD45^+^ KUL01^+^ cells) from caecal tissues were sorted from non-infected, infected, conventional, GF chickens and infected GF chickens receiving a microbiota from healthy conventional chickens and a high-throughput qPCR was performed on cDNA. We focused on genes associated to pro- and anti-inflammatory processes (cytokines, chemokines) and pattern recognition receptors (PRRs) such as TLRs (77 genes in total). In non-infected chickens, the transcriptomic profile of sorted KUL01^+^ cells was significantly different between conventional and GF chickens (Additional file 2: Figure S2). In non-infected conventional chickens compared to GF chickens, 4 genes were found upregulated (*peroxisome proliferator-activated receptor* γ (*PPARγ), collectin subfamily member 12 (COLEC12), CD80* and *Toll Like Receptor* (*TLR)4*) and 2 genes downregulated (*Aconitate Decarboxylase 1 (ACOD1)* and *Signaling Lymphocytic Activation Molecule Family Member 1 (SLAMF1)*) suggesting that microbiota modifies macrophage gene expression at homeostasis. In a heatmap representation of macrophage gene expression from non-infected, infected conventional, GF and GF chickens given microbiota, 3 different clusters were identified to be different with the infection: genes upregulated in a microbiota dependent manner (cluster 1), genes upregulated independently of microbiota (cluster 2), genes downregulated independently of microbiota (cluster 3) (Fig. 3). At day 7 pi, a significant upregulation of 15 genes (*Suppressor of cytokine signaling protein 1* (*SOCS1), TLR15, TLR5, IL1β, TNF superfamily member 13b (TNFS13B), RANTES (CCL5), CXCL13, IL8L1 (CXCLi1), TLR5, IL10, IL12p40, Colony Stimulating Factor (CSF) 3, ACOD1, Nitric Oxide Synthase 2 (NOS2)* and *IL6*) was observed in infected conventional chickens compared to their non-infected counterparts (Fig. 4A *left panel,* B). Only 2 genes, *ACOD1* and *NOS2* were upregulated significantly in GF-infected chickens compared to non-infected chickens but at a lower level (twofold increase in GF *versus* fourfold increase conventional chickens) than in conventional infected chickens (Fig. 4A *right panel,* B). Activated immune cells display high *ACOD1* and *NOS2* gene expression [32, 33] suggesting that macrophages are activated in *E. tenella* infection independently of microbiota. Thirty-eight genes were downregulated in macrophages from GF-infected chickens while only 28 genes were found downregulated in macrophages from conventional infected chickens compared to non-infected chickens (Fig. 4A). Twenty-five genes were found to be downregulated in macrophages from both conventional and GF-infected chickens (*Chemokine Receptor (CCR) 7, CCR6, CD74, COLEC12, CD38, CD86, CLEC17A, CSF1R, Interferon Alpha And Beta Receptor Subunit (IFNAR) 1, IFNRA2, Interferon Gamma Receptor (IFNGR) 1, Glutamate-Ammonia Ligase (GLUL), IL4R, MER Proto-Oncogene Tyrosine Kinase (MERTK), MYD88, Interferon Regulatory Factor 4 (IRF4), mannose receptor C-type 1 like B (MRCL1B), Notch Receptor 2 (NOTCH2), ODC, PPARγ, Signal Transducer And Activator Of Transcription (STAT)3, STAT6, Transforming Growth Factor (TGF)β, TIMD4), TLR7*). These genes are mostly receptors (i.e.: *CCR6*, *CCR7, CSF1R, Interferon Alpha* and *Beta Receptor Subunit IFNAR1, IFNRA2, IFNGR1, IL4R, …)* and transcription factors (i.e.: *NOTCH2, STAT3, STAT6*, …) (Fig. 4B). Three genes *(DEC205*, *CD150/SLAMF1*) and *CD180* were downregulated only in conventional infected chickens. Thirteen genes including *Prostaglandin-Endoperoxide Synthase 2* (*PTGS2), CD80, CD25, CSF1, CD44, CSF3R, I18L2 (CXCLi2), Phospholipase A2 Receptor 1 (PLA2R1), TLR4, Tumor Necrosis Factor (TNF) α, Lymphocyte Antigen (LY) 86, LY96* and *Vascular Endothelial Growth Factor A (VEGFA)* were only downregulated in GF-infected chickens. In this group, several inflammatory mediator gene expressions (*PTGS2, CSF1, IL8L2* and *TNFα*) were downregulated in GF-infected chickens wheras no inflammatory mediator was downregulated in conventional infected chickens.

### Microbiota promotes macrophage pro-inflammatory gene expression in *E. tenella* infection

**Fig. 5:**
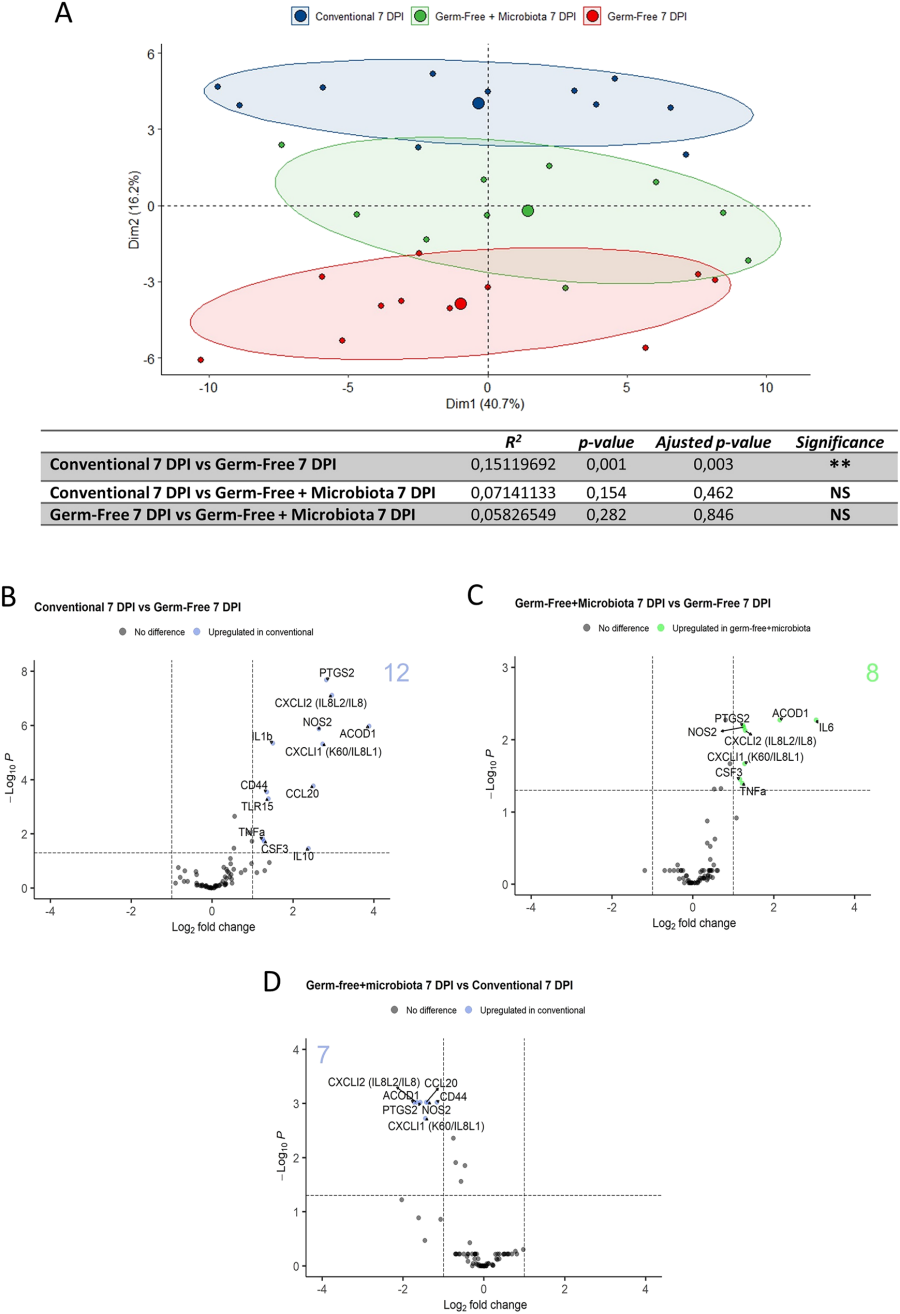
Microbiota promotes macrophage inflammatory response. **A** Principal component (PCA) analysis of macrophage transcriptomic profiles compared at 7 days pi between cells from conventional (blue), GF (red) and GF chickens receiving a microbiota from conventional chickens at 4 days pi (green). PERMANOVA statistical test result is showed on the upper right corner and in the table. *P < 0.05. Each point on PCA plots represents an animal and a larger dot corresponds to the barycenter according to each group. Dim1 axis and Dim2 axis show principal components 1 and 2 and the percent variation corresponding to each component are shown in parenthesis. Differential gene expression was determined for infected conventional *versus* (*vs*) infected GF (**B**), infected GF + microbiota *vs* infected GF (**C**) and infected GF + microbiota *vs* infected conventional (**D**), infected GF + microbiota) and are represented as volcano plots. Scattered dots represent each gene. Only significantly different gene expression (P < 0.05) and fold change > 2 or < -2 are indicated. These thresholds are indicated with dashed lines on the plots

Comparison of macrophage transcriptome profiles from infected chickens shows a distinct transcriptomic profile between conventional and GF chickens suggesting a role of the microbiota in these responses. The addition of microbiota at 4 days pi to infected GF chickens led to an intermediate transcriptomic profile (Fig. 5A). To further investigate the impact of microbiota on macrophage responses in *E. tenella* infection, we performed a differential analysis of gene expression within infected groups (Fig. 5B–D). Differential gene expression between conventional and GF-infected chickens led to the identification of genes that were significantly up-regulated in the presence of microbiota: *TNFα, CSF3, IL10, TLR15, CD44, CCL20, IL1β, IL8L1 (CXCLi1), IL8L2 (CXCLi2), NOS2, ACOD1* and *PTGS2* (Fig. 5B). The administration of microbiota to GF-infected chickens induced the expression of *PTGS2, ACOD1, IL8L1 (CXCLi1), I18L2 (CXCLi2), NOS2, CSF3, TNFα* and *IL6* (Fig. 5C). Comparison between GF-infected chickens receiving a microbiota and conventional infected chickens indicated that *PTGS2, ACOD1, IL8L1 (CXCLi1), IL8L2 (CXCLi2), NOS2, CD44* and *CCL20* were significantly up-regulated to a similar level to conventional chickens (Fig. 5D), indicating a role for microbiota in restoring the macrophage response. Moreover, the macrophage inflammatory response described in conventional chickens was already present at 3.5 days pi (Additional file 3: Figure S3) at a time point for which the epithelial layer was not yet damaged. However, the macrophage response was more exacerbated at 7 days pi when the epithelium was severely damaged, suggesting a role for tissue-infiltrating microbiota in this process.

## Discussion

In this study, we investigated the involvement of the microbiota on macrophage infiltration and response during *E. tenella* infection. Using an original model of conventional and GF broilers, the results highlight that, in *E. tenella* infection, the caecal microbiota enhances macrophage recruitment and the inflammatory response.

During *E. tenella* infection, an infiltration of leukocytes such as macrophages, heterophils or lymphocytes has been demonstrated [3, 6, 34, 35]. In *E. bovis* infection, the number of macrophages has also been found to be increased [7]. As highlighted previously [18], mucosal thickness and leukocyte infiltration are increased in conventional chickens at 7 days pi but not in GF chickens. Here, we have identified, for the first time, that macrophage recruitment requires the presence of microbiota and that *E. tenella* infection alone is not sufficient to induce this process. In support of this conclusion, the addition of microbiota to GF-infected chickens partially enabled macrophage recruitment as observed in conventional animals but not in GF chickens and, confirms the key role of microbiota in macrophage recruitment during *E. tenella* infection. Therefore, among leukocytes, infection-associated macrophage infiltration may be partly responsible for the mucosal thickness observed in the presence of microbiota. It is already known that the microbiota plays an important role in the maturation of the immune system at homeostasis, and that resident intestinal macrophages respond poorly to inflammation [36]. However, when intestinal integrity is impaired, the inflammatory response leads to the recruitment of blood monocytes, which differentiate in the tissues into macrophages capable of releasing large quantities of inflammatory mediators that may be responsible for tissue damages [36]. Following infection and during the repair process, macrophages may play a beneficial role in reducing inflammation [37]. With this in mind, at the early stages of *E. tenella* infection, when the integrity of the epithelium is not yet damaged, there is no increase in macrophages with the microbiota. At a more advanced stage of infection, the integrity of the intestinal barrier is impaired, leading to infiltration of the microbiota, which further increases the number of macrophages with a pro-inflammatory profile in the caecal tissues. We could postulate that after the peak of inflammation, macrophage may change their response towards an anti-inflammatory profile to favor tissue repair.

During *E. tenella* infection, macrophages show a stronger pro-inflammatory response in the presence of microbiota. Genes such as *TNFα, IL10, TLR15, IL8L1 (CXCLi1), IL8L2 (CXCLi2), NOS2, ACOD1* and *PTGS2* were upregulated with infection and microbiota, suggesting that macrophages may largely participate in the pro-inflammatory response described in caecal tissues [4, 5, 18, 34, 35]. During *E. tenella* infection, macrophages express *IL8* in the presence of microbiota, suggesting that, in addition to IL8-producing intestinal epithelial cells, IL8-producing macrophages may participate in part in the recruitment of immune cells such as heterophils to the site of infection [38]. Among other mediators produced by macrophages, prostaglandins are synthetized by the PTGS2 enzyme during inflammation [39]. Prostaglandins display a key role in inflammatory processes facilitating the recruitment of leukocytes to sites of inflammation by increasing of vascular permeability and blood flow [40]. In our model, we previously showed that *PTGS2* was up-regulated during *E. tenella* infection in a microbiota-dependent manner in caecal tissues [18]. Here, similar results were observed in caecal macrophages suggesting that they may be one of the main cells expressing this enzyme. It is therefore possible that prostaglandins are partly responsible for the genesis of caecal lesions in conventional chickens infected with *E. tenella* infection. In accordance with this hypothesis, a study using a high dose of a nonsteroidal anti-inflammatory drug that inhibits prostaglandin synthesis led to partially lower lesion scores in chicken coccidiosis [41]. Similarly, protease-activated receptors knockout mice infected with another apicomplexan parasite, *T. gondii,* showed a decrease in innate inflammatory mediators such as IL6, chemokines and prostaglandin E2 and a reduction in macroscopic lesions [42]. Macrophages could therefore be strongly involved in the genesis of caecal lesions during *E. tenella* infection**.** To test this hypothesis, it would be necessary to use genetically modified chickens depleted in macrophages. The absence of this tool led us to assess macrophage depletion using clodronate but this method was not successful in our hand. Therefore, a murine model of macrophage depletion such as the one developed by Schreiber et al. [43] may be useful to study the role of macrophages in the physiopathology of *Eimeria* infection.

In *E. tenella* infection, the *ACOD1* and *NOS2* genes are highly expressed with microbiota but are also increased at a lower level in the absence of microbiota suggesting that macrophages are activated in both models. ACOD1 is involved in immune responses by producing itaconate and reactive oxygen species (ROS) [32]. NOS2 leads to the synthesis of a free radical nitric oxide (NO). ROS including NO have antimicrobial properties [44]. However, depending on the pathogen, ACOD1 expression can lead to beneficial [45] or deleterious effects [46]. Indeed, NO production could also have negative effects and lead to tissue damage involved in various diseases [47]. In the case of *E. tenella* infection, high expression of *ACOD1* and *NOS2* may be deleterious, as lower expression of these enzymes in GF-infected chickens results in fewer caecal lesions [18]. Therefore, adequate expression of ACOD1 and NOS2 in macrophages may be necessary to control the parasite without inducing damage to host tissues. Further studies are needed to investigate their role in the physiopathology of *E. tenella* infection.

While expressing highly inflammatory mediators, macrophages also express anti-inflammatory mediators such as IL10 during infection. Previous studies have described an increase of *IL10* in chickens infected with different *Eimeria* species [5, 48, 49]. As an anti-inflammatory cytokine, IL10 controls host immune response by inhibiting pro-inflammatory cytokines, preventing, repairing host cell damage and maintaining intestinal homeostasis and integrity [50–53]. In *Eimeria* infection, the use of an anti-IL10 antibody resulted in no difference in the excretion of *Eimeria* oocysts [54], suggesting that IL10 may have a modulatory role on inflammation and tissue damage rather than a direct effect on the parasite. T cells (mainly regulatory T cells) are known to be the main source of IL10 during protozoan infections. However, macrophages can also contribute to IL10 secretion [55]. We have previously described that the microbiota promotes an increase in *IL10* gene expression in caecal tissue in *E. tenella* [18]. In the present study, our data suggest that microbiota enhances *IL10* gene expression in macrophages during *E. tenella* infection. Macrophages may then represent a source of IL10 modulating the inflammatory response and facilitating tissue repair. This dichotomy of macrophage pro- and anti-inflammatory responses may be based on the presence of different macrophage subpopulations in the caeca similarly to mammals [56]. Further studies would be required to determine the macrophage subpopulations in the caecum.

This study also indicates that a number of genes were downregulated with infection independently of the microbiota. These genes are mainly receptors and transcription factors suggesting that, although the inflammatory response of macrophages is high, cellular signaling pathways are modified by infection, independently of the microbiota, probably via the host epithelial cells in which it develops, resulting in the expression of molecules down-regulating the macrophage response to allow its development. Another hypothesis could support the immune system’s negative self-regulation to prevent exacerbated inflammation. These opposite effects have already been reported in *T. gondii* infection of macrophages, in which TLR pathways inducing the expression of pro-inflammatory mediator are downregulated [57, 58]. The precise signaling mechanisms involved in this regulation appear to be diverse, ranging from inhibition of nuclear translocation of transcriptional factors and miRs to epigenetic regulations [57, 59, 60]. It is therefore possible that similar mechanisms are observed in *E. tenella* infection.

**Fig. 6:**
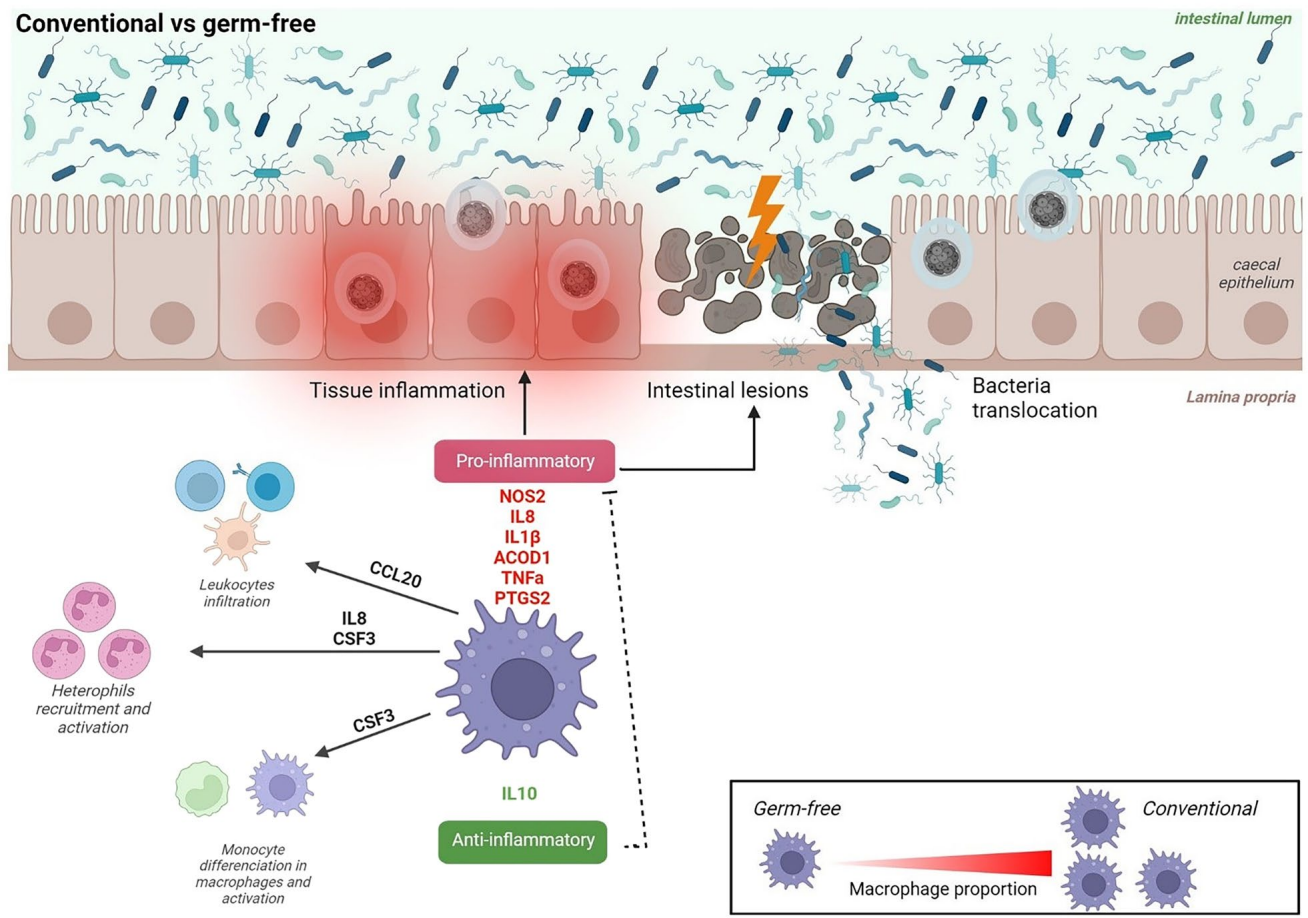
Summary of microbiota impact on caecal macrophage response to *E. tenella* infection. Microbiota promotes macrophage recruitment on the site of infection and a pro-inflammatory response associated to the formation of caecal lesions. It may then be possible that macrophage pro-inflammatory response plays a role in the physiopathology of *E. tenella* infection. Created with BioRender.com (Agreement number: OO25YBF87R)

As summarized in Fig. 6, microbiota promotes macrophage recruitment, activation and the modulation of various signalling pathways in *E. tenella* infection. Pro-inflammatory genes are mainly induced in the presence of microbiota which could potentially lead to the genesis of caecal lesions. Strategies aimed at modulating the microbiota would be of interest to attenuate the inflammatory status of macrophages and the consequences on the physiopathology of *E. tenella* infection.

### Supplementary Information

**Additional file 1: Figure S1. **MHCII and KUL01 staining results in only one macrophage population in chicken caeca. Caecal cells and splenocytes were isolated from 3 weeks old Ross PM3 chickens. Debris were removed based on FSC and SSC. Live cells were selected using a live/dead marker, Zombie Aqua™. Dotplot of CD45^+^ KUL01^+^ with MHCII Alexa fluor A488 staining for caeca cells (A) and splenocytes (B) resulting in one KUL01^+^ population in caeca and two KUL01^+^ subsets in spleen (MHCII^high^ KUL01^low^ and MHCII^low^ KUL01^high^).

**Additional file 2: Figure S2. **Macrophage transcriptomic profile is different between non-infected conventional and GF chickens. Principal component (PCA) analysis of macrophage transcriptomic profiles from non-infected conventional (blue) and GF (red) chickens. PERMANOVA statistical test result is showed on the upper right corner. *P < 0.05. Each point on PCA plots represents an animal and a larger dot corresponds to the barycenter according to each group. Dim1 axis and Dim2 axis show principal components 1 and 2 and the percentage variation corresponding to each component are shown in parenthesis. Differential gene expression was determined for non-infected conventional vs GF and represented as volcano plot. Scattered dots represent each gene. Only significantly different gene expression (P < 0.05) and fold change > 2 or < -2 are indicated. These thresholds are indicated with dashed lines on the plots.

**Additional file 3: Figure S3. **Macrophage transcriptomic profile in conventional and GF chickens during a kinetic of infection. NI and DPI represent non-infected and days pi chickens, respectively. Conventional chickens are indicated in blue, GF chickens in red and GF chickens that received a conventional microbiota in green. Delta Ct gene expression was normalized using a Z-score for each gene. Gradient color from blue to yellow indicated low level and high level expression respectively. A hierarchical clustering was performed following Z-score for each gene and clusters are indicated in the left of the heatmap. (n ≥ 8 chickens / group).

**Additional file 4: Table S1**. List of primers used for Fluidigm.

## Data Availability

The raw data supporting the conclusions of this article will be made available by the authors, without undue reservation.
