# Supplementary figures and images for: Microbiota promotes recruitment and pro-inflammatory response of caecal macrophages during E. tenella infection
Source: Gut Pathog. 2023 Dec 14;15:65. doi: 10.1186/s13099-023-00591-8 (PMC10720127; doi:10.1186/s13099-023-00591-8)

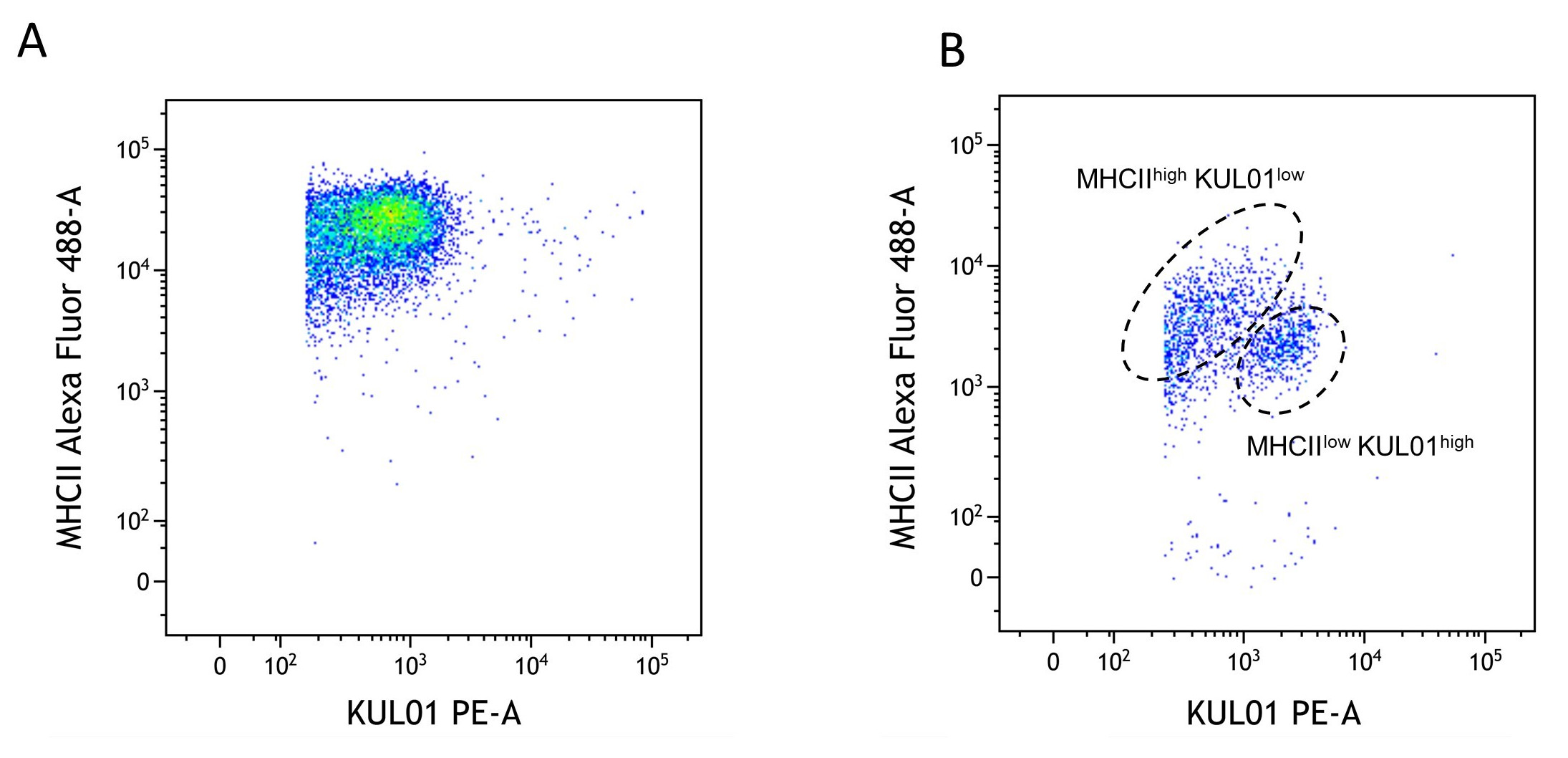

Supplement: Supplementary file 1 — Additional file 1: Figure S1. MHCII and KUL01 staining results in only one macrophage population in chicken caeca. Caecal cells and splenocytes were isolated from 3 weeks old Ross PM3 chickens. Debris were removed based on FSC and SSC. Live cells were selected using a live/dead marker, Zombie Aqua™. Dotplot of CD45+ KUL01+ with MHCII Alexa fluor A488 staining for caeca cells (A) and splenocytes (B) resulting in one KUL01+ population in caeca and two KUL01+ subsets in spleen (MHCIIhigh KUL01low and MHCIIlow KUL01high). [file 13099_2023_591_MOESM1_ESM.jpg]

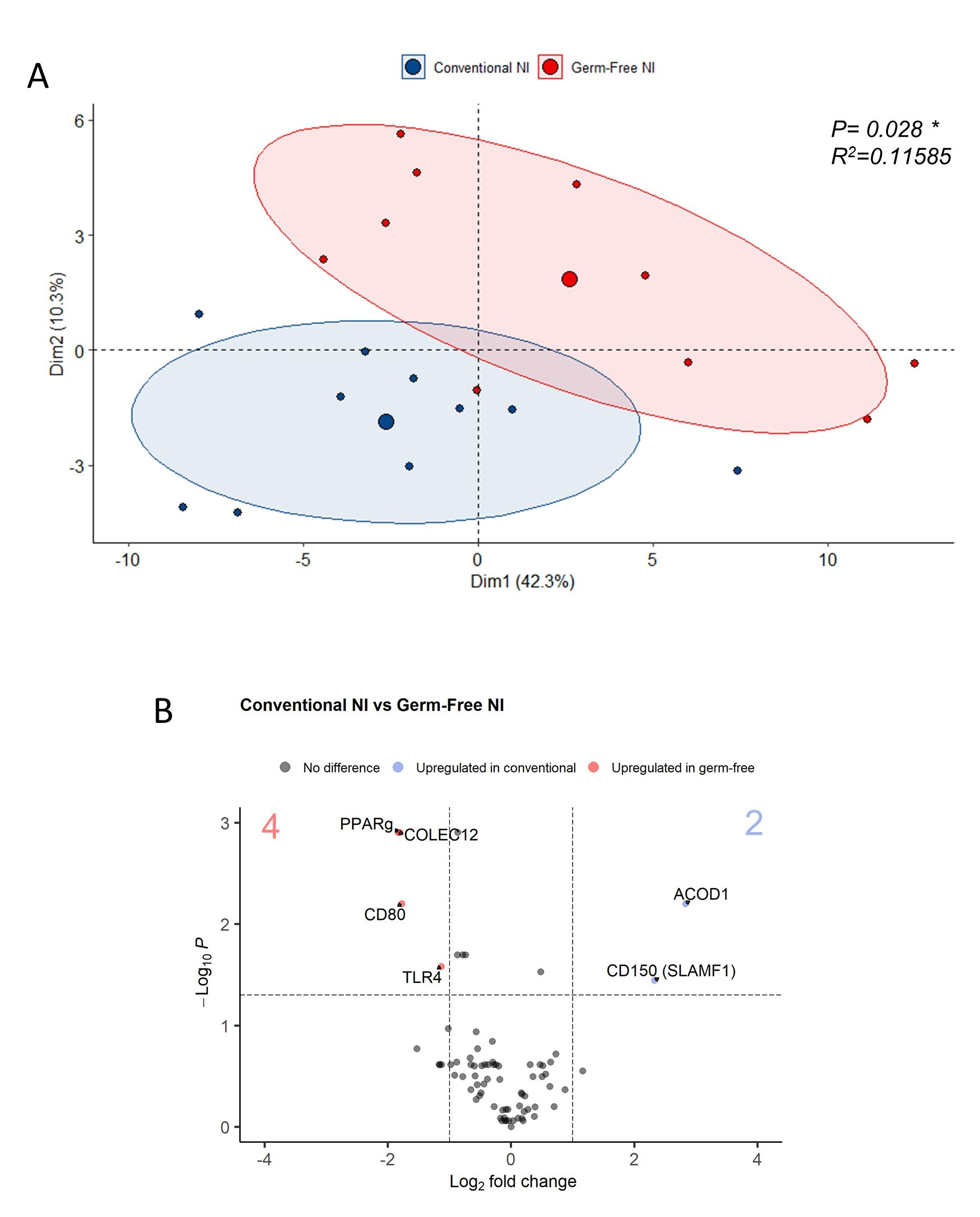

Supplement: Supplementary file 2 — Additional file 2: Figure S2. Macrophage transcriptomic profile is different between non-infected conventional and GF chickens. Principal component (PCA) analysis of macrophage transcriptomic profiles from non-infected conventional (blue) and GF (red) chickens. PERMANOVA statistical test result is showed on the upper right corner. *P < 0.05. Each point on PCA plots represents an animal and a larger dot corresponds to the barycenter according to each group. Dim1 axis and Dim2 axis show principal components 1 and 2 and the percentage variation corresponding to each component are shown in parenthesis. Differential gene expression was determined for non-infected conventional vs GF and represented as volcano plot. Scattered dots represent each gene. Only significantly different gene expression (P < 0.05) and fold change > 2 or < -2 are indicated. These thresholds are indicated with dashed lines on the plots. [file 13099_2023_591_MOESM2_ESM.jpg]

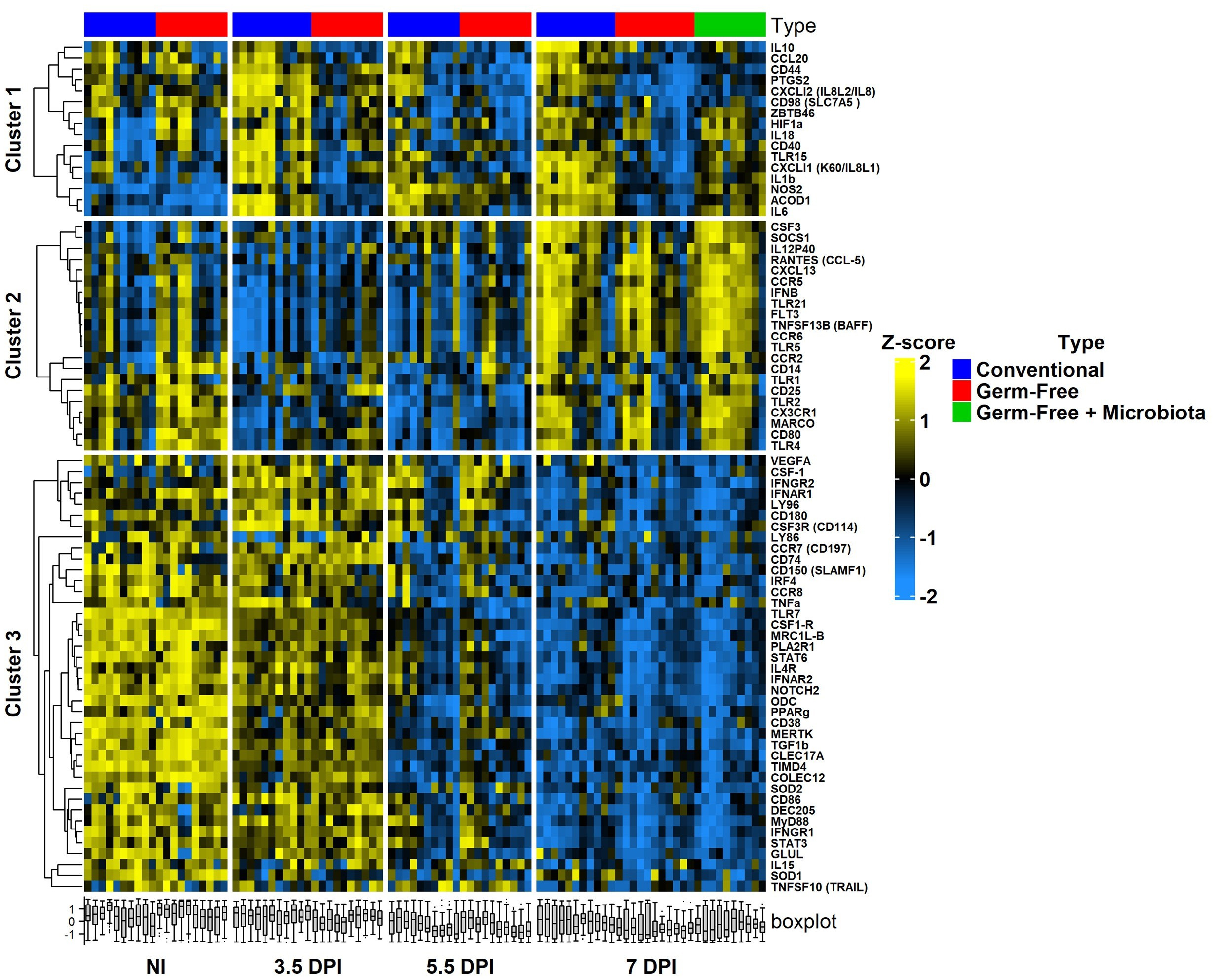

Supplement: Supplementary file 3 — Additional file 3: Figure S3. Macrophage transcriptomic profile in conventional and GF chickens during a kinetic of infection. NI and DPI represent non-infected and days pi chickens, respectively. Conventional chickens are indicated in blue, GF chickens in red and GF chickens that received a conventional microbiota in green. Delta Ct gene expression was normalized using a Z-score for each gene. Gradient color from blue to yellow indicated low level and high level expression respectively. A hierarchical clustering was performed following Z-score for each gene and clusters are indicated in the left of the heatmap. (n ≥ 8 chickens / group). [file 13099_2023_591_MOESM3_ESM.jpg]
